# Strangulated Hernia Treated by Exhaustion through the Elastic Tube

**Published:** 1843-07

**Authors:** C. S. Webber

**Affiliations:** Orford


*Strangulated Hernia Treated by Exhaustion through the Elastic Tube.*—By C. S. Webber, Esq., Orford.—Mr. Webber of Orford, relates a case where he succeeded in the way recommended by Mr. Maunder; that he endeavored to produce a vacuum below the stricture by the exhausting power of the syringe. The tumor, which occupied the scrotum, was of the size of a goose's egg, remarkably tense and unyielding, with all the syptoms of strangulation. The taxis was diligently and carefully applied for about twenty minutes without succcess; the patient was then bled to syncope, and the taxis again had recourse to, followed by the application of cold to the tumor, and such other remedies as circumstances permitted. Before having recourse to an operation, Mr. Webber resolved to try Mr. Maunder's plan, which he did as follows:

A common enema having been previously administered, I introduced the œsophagus tube of Weiss's stomach-pump with tolerable facility, until it arrived (as nearly as I could judge) at the sigmoid flexure of the colon, where it encountered some degree of resistance, which yielded, however, in a short time, to moderate pressure, after the injection of a little cold water. The tube was now passed gradually up until its whole length, about twenty-five inches, had been introduced, the brass extremity alone remaining without.

After waiting a quarter of an hour, between two and three pints of water were thrown up and retained in the bowel for a short period; the cylinder of the pump having been unscrewed from the elastic tube, and the mouth of the latter closed by the thumb, on withdrawing which the fluid repassed in a jet of considerable force. The cylinder was now readjusted to the tube, and the action of the machine being reversed, the piston was worked rapidly with the view of producing a degree af exhaustion or partial vacuum in the intestine; gentle taxis was at the same time resumed, and after the expiration of four or five minutes (during the latter part of which the patient complained of a sensation of bearing down referred to the whole abdominal region), I had the satisfaction to feel the contents of the tumor recede from beneath my fingers, and slip into the abdomen with the usual gurgling which accompanies the return of intestine.

The man recovered without an untoward symptom, and was removed to his father's house at Wickham, a distance of about nine miles, on the third day afterwards.

I am disposed to attribute the successful result of the foregoing case, in a principal degree, to the production of a partial vacuum in the intestine immediately below the seat of strangulation, by the exhausting process, that being the direct agent to which the obstruction appears to have yielded; and I am induced to recommend a trial of this measuse to the profession as an adjuvant to that valuable instrument, the elastic tube, in cases where the latter alone may prove inefficient.

I have since reperused Dr. O'Beirn's excellent work on Defæcation, with the view of ascertaining whether exhaustion through the tube had been adopted in any of his cases. I cannot discover that the doctor has availed himself of this expedient, although a case of constipation is cited by him in which Dr. Duguid, of Kirkwall, in Scotland, introduced an elastic tube into the colon with the effect of discharging an accumulation of fæces; and I observe that this gentleman attempted to exhaust with the view of overcoming the resistance he experienced to the passage of the tube at the sigmoid flexure, but unsuccessfully, which circumstance induced him to undervalue the measure. There is, however, another case which seems to have suggested to Dr. Duguid the use of the tube, and which is reported in the Medical and Physical Journal, for December, 1827, being communicated by Dr. James Johnson, as having occurred in February, 1826, in the practice of Dr. Alexander, of Genoa, who appears to have employed exhaustion through a hollow bougie, after twelve days of constipation, and the failure of tobacca enemata and various other remedies, with complete success. This case, which is a very interesting one, seems to have excited but little attention.

I cannot conclude without expressing my conviction (in nearly the words of Dr. O'Beirne, who uses them, however, with reference to the tube only), that no surgeon is justified in proceeding to the operation for strangulated intestinal hernia, without having previously given a fair trial to the measures above alluded to.—*Braithwaite's Retrospect, Part the Sixth*, from *London Lancet*, May 28, 1842.

